# Myosin light-chain phosphatase regulates basal actomyosin oscillations during morphogenesis

**DOI:** 10.1038/ncomms10746

**Published:** 2016-02-18

**Authors:** Andrea Valencia-Expósito, Inna Grosheva, David G. Míguez, Acaimo González-Reyes, María D. Martín-Bermudo

**Affiliations:** 1 Centro Andaluz de Biología del Desarrollo, Universidad Pablo de Olavide/CSIC/JA, Carretera de Utrera km 1, Sevilla 41013, Spain; 2 Departamento de Física de la Materia Condensada, Instituto de Ciencias de Materiales Nicolás Cabrera, Condensed Matter Physics Center (IFIMAC), Universidad Autónoma de Madrid, Madrid 28049, Spain

## Abstract

Contractile actomyosin networks generate forces that drive tissue morphogenesis. Actomyosin contractility is controlled primarily by reversible phosphorylation of the myosin-II regulatory light chain through the action of myosin kinases and phosphatases. While the role of myosin light-chain kinase in regulating contractility during morphogenesis has been largely characterized, there is surprisingly little information on myosin light-chain phosphatase (MLCP) function in this context. Here, we use live imaging of *Drosophila* follicle cells combined with mathematical modelling to demonstrate that the MLCP subunit *flapwing* (*flw*) is a key regulator of basal myosin oscillations and cell contractions underlying egg chamber elongation. Flw expression decreases specifically on the basal side of follicle cells at the onset of contraction and *flw* controls the initiation and periodicity of basal actomyosin oscillations. Contrary to previous reports, basal F-actin pulsates similarly to myosin. Finally, we propose a quantitative model in which periodic basal actomyosin oscillations arise in a cell-autonomous fashion from intrinsic properties of motor assemblies.

## Abstract (web summary)

Actomyosin contractility is regulated by phosphorylation of myosin regulatory light chain; much of the work in this area has focused on the kinase. Here the authors use *Drosophila* follicle cells and modelling to show that the phosphatase subunit Flapwing controls the initiation and dynamics of actomyosin oscillations.

## Graphical abstract

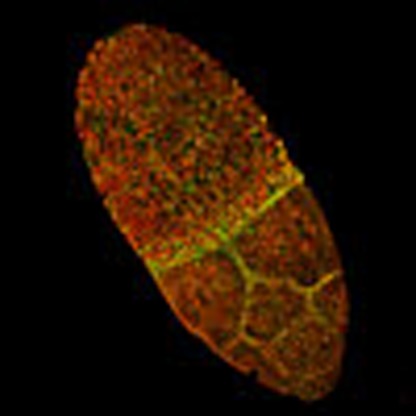

Actomyosin-driven cell contractility is indispensable during morphogenesis for the acquisition of the three-dimensional shape of tissues and organs^1^. It relies on forces generated by the contraction of an actin filament network through the activity of the motor protein non-muscle myosin-II (Myo-II)^2^. Myo-II molecules are hexameric enzymes consisting of two heavy chains, two regulatory light chains (MRLC) and two essential light chains^3^. Myo-II activity is regulated by dynamic phosphorylation and dephosphorylation of the MRLC^4^. Phosphorylation of MRLC by several kinases brings the myosin complex into an active state, thus allowing its interaction with actin filaments and the generation of contractile force. On the contrary, myosin light-chain phosphatase (MLCP), a heterotrimer consisting of a Ser/Thr phosphatase catalytic subunit (PP1c), a myosin phosphatase targeting subunit (MYPT1/2) and a 20-kDa small subunit, performs the opposite role, myosin inactivation via MRLC dephosphorylation^5^. Remarkably, while there is a great deal of information concerning the role of MRLC kinases on the regulation of cell contractility during morphogenesis, the function of MRLC phosphatases in this context remains largely unknown.

Contractility-based morphogenetic transitions require the local activation of actomyosin networks in particular groups of cells at specific developmental time points. In contrast to the detailed characterization of apical contractility, the molecular and mechanical mechanisms underlying basal actomyosin contractility during morphogenesis are less known. One well-studied example of basal cell contraction essential for tissue morphogenesis is that of the follicle cells (FCs) during egg formation in the adult *Drosophila* ovary. In oogenesis, newly formed egg chambers (or follicles) composed of 16-germline cysts surrounded by a monolayer follicular epithelium go through 14 developmental stages (from S1 to S14) to eventually give rise to mature eggs, which are elongated anterior–posteriorly^6^. Egg elongation takes place from S5 to late S10 and it relies on two temporally distinct phenomena. First, during the initial stages of egg elongation, egg chamber global rotation helps create a ‘molecular corset' of organized extracellular matrix that biases growth along its anterior–posterior axis^7^. Second, periodic basal contractions driven by myosin oscillations that initiate at S9 eventually shape the mature egg^8^. Recently, the oscillatory behaviour of myosin contractile activity in FCs has been proposed to originate from pressure exerted by the underlying growing germline. In this mechano-chemical model, pressure from the germline would activate the Rho-kinase (RhoK) pathway, thus inducing myosin assembly and contraction in FCs^9^. However, the signals that trigger formation of basal contractile arrays at specific time points of egg chamber development are still undefined.

Here, we identify *flapwing (flw)*, which codes for one of the four PP1c subunits that exist in *Drosophila*, as a key regulator of basal contractile activity in FCs. We show that at S9, concomitant with the onset of basal contraction, Flw expression decreases specifically on the basal side of FCs. In addition, we find that *flw* is required for the initiation and periodicity of basal myosin oscillations. In contrast to previous observations^8^, we demonstrate that basal F-actin undergoes periodic pulsations similarly to myosin. Based on these observations and using *in silico* modelling, we propose that cell-autonomous basal actomyosin oscillations governing egg chamber elongation arise from the intrinsic properties of motor assemblies.

## Results

### Dynamic distribution of Flw in FCs during oogenesis

**Figure 1:**
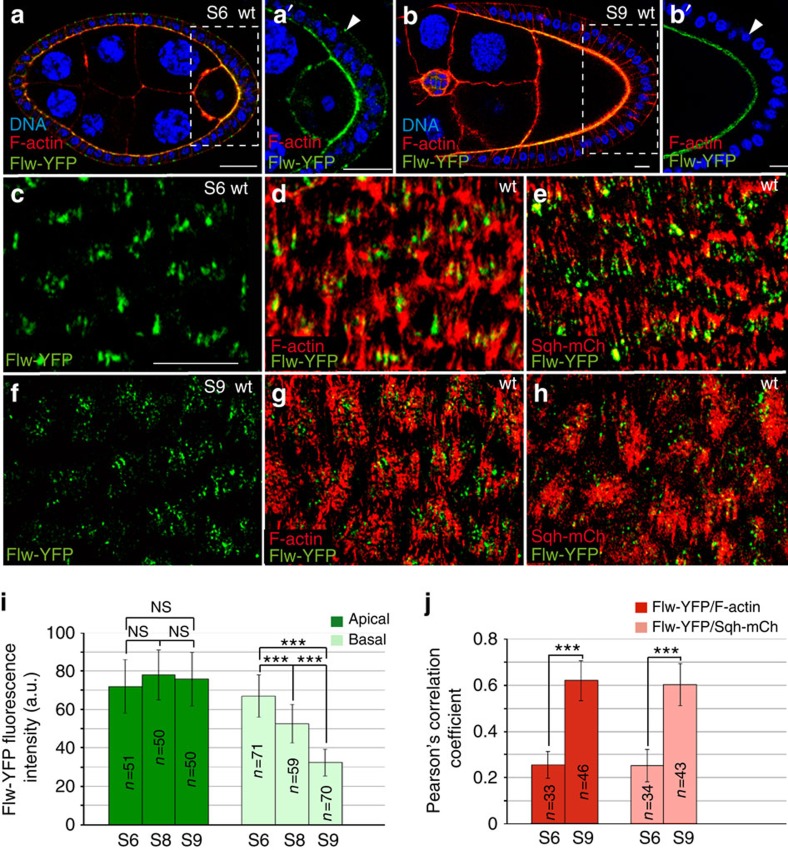
Control egg chamber showing the localization of Flw-YFP (green), DNA (blue) and F-actin or myosin regulatory light chain (Sqh) in red. (**a**, **b**) Sagittal planes of S6 (**a**) and S9 (**b**) egg chambers stained with Rhodamine-Phalloidin to visualize F-actin and the nuclear marker TO-PRO-3. (**a**) At S6, Flw-YFP is expressed at both the apical and basal sides of FCs. (**b**) At S9, basal Flw-YFP localization is specifically reduced. (**a**′, **b**′) Magnifications of the white boxes in **a** and **b**, respectively. (**c**–**h**) Basal surface views of S6 (**c**–**e**) and S9 (**f**–**h**) follicles expressing Flw-YFP and either stained with Rhodamine-Phalloidin to detect F-actin (**d**, **g**) or expressing Sqh-mCherry to visualize myosin (**e**, **h**). (**d**) At S6, Flw-YFP largely co-localizes with F-actin, distributing asymmetrically on the filaments. (**e**) At S6, Flw-YFP and Sqh-mCherry show a non-overlapping distribution. (**g**,**h**) At S9, faint basal Flw-YFP puncta co-localize with actomyosin filaments. (**i**) Quantification of the apical and basal levels of Flw-YFP at S6, S8 and S9. (**j**) Pearson's coefficient correlation between Flw-YFP and F-actin and Flw-YFP and Sqh-mCh at S6 and S9. The statistical significance of differences was assessed with a *t*-test. NS, not significant, *** *P*<0.0001. All errors bars indicate s.d. Scale bar, 10 mm. Mean of *n*=15 egg chambers, assessed over five independent experiments.

To analyse Flw expression in FCs we used the protein-trap line *flw-YFP-159*, which codes for the Flw-YFP protein^10^. The distribution of Flw-YFP throughout oogenesis was dynamic. Thus, from S5 to S8, Flw-YFP was localized in dot-like structures both at the apical and basal domains of FCs (Fig. 1a,c,d). In addition, Flw-YFP localization on the basal side decreased from S6 to S9, while that on the apical surface remained unchanged (Fig. 1a,b). To better characterize these changes in Flw-YFP accumulation, we quantified the fluorescence intensity of Flw-YFP, as a function of the surface area, at the basal and apical sides of S6, S8 and S9 FCs (at least eight different egg chambers were scored in each case). These measurements showed that, while the apical levels of Flw-YFP were maintained throughout S6–S9, the basal levels were indeed reduced by 20% from S6 to S8 and by half from S6 to S9, when oscillations started (Fig. 1i). Next, we decided to correlate Flw-YFP localization with that of basal actomyosin fibres. To this end, we performed co-localization studies of Flw-YFP and F-actin (visualized with TRITC-phalloidin) or myosin (using the red fluorescent protein mCherry fused to *spaghetti squash*, *sqh*, the gene encoding for the *Drosophila* non-muscle Myo-II regulatory light chain^11^). Organization of basal actin bundles during oogenesis is quite dynamic. In the germarium, they show a circumferential organization perpendicular to the anterior–posterior axis. This tissue level alignment is then maintained and reinforced up to S10A, when actin bundles are more apparent and look like stress fibres^12^ (Fig. 1g). At S10B, actin fibres thicken and by S11 adopt a fan-shaped morphology and change their orientation by 90^o^. At S12, normal orientation is restored^12^. We found that while at S6, Flw-YFP seemed to localize to the tips of the F-actin and Sqh-mCherry bundles (Fig. 1d,e), at S9 it was found distributed along the F-actin and myosin bundles in a dotted pattern (Fig. 1g,h). To analyse this in more detail, we quantified the level of co-localization of the different markers using the Pearson's correlation coefficient (PCC, Materials and methods section, Fig. 1j). This analysis revealed reduced co-localization of Flw-YFP with F-actin or Sqh-mCherry at the tip of the bundles at S6 (PCC=0.25 and 0.24, respectively) and a stronger association along the bundles at S9 (PCC=0.62 and 0.60, respectively).

In summary, analysis of Flw-YFP localization during oogenesis showed that while basal Flw-YFP levels are high from S5 to S8, before basal myosin oscillations appear, they decrease dramatically by S9, coinciding with the onset of basal myosin fluctuations and cell contractions.

### Flw regulates the assembly of basal contractile structures

**Figure 2:**
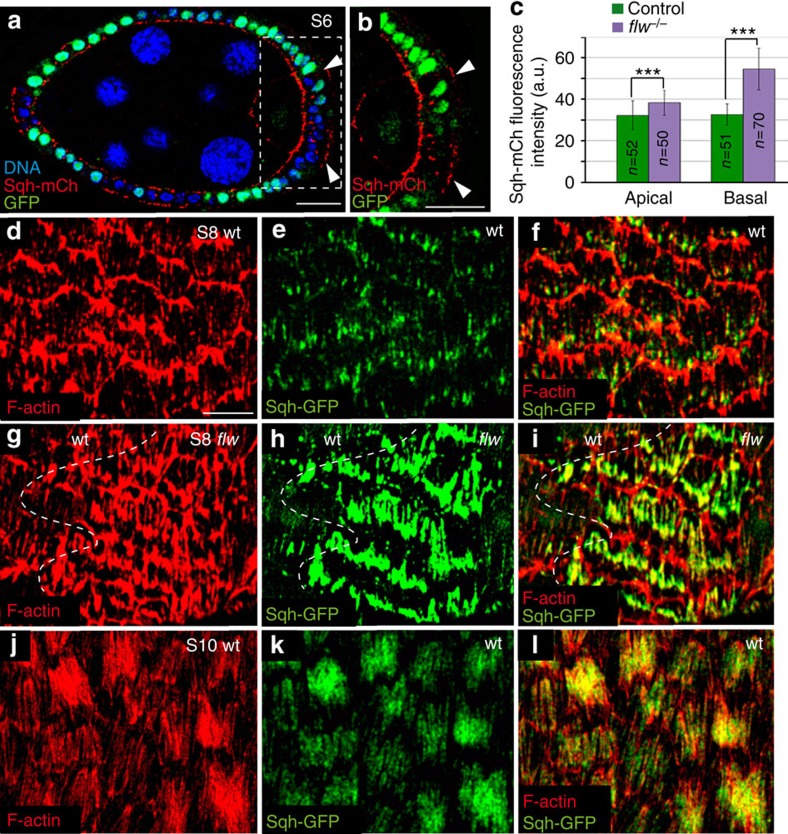
Loss of *flw* enhances basal recruitment of actin and myosin. (**a**) Sagittal plane of a mosaic S8 follicle expressing Sqh-mCherry (red) and containing *flw* mutant clones stained for anti-GFP (green) and the nuclear marker TO-PRO-3 (blue). *flw* mutant FCs (labelled by the absence of GFP, arrowheads) show higher levels of basal Sqh-mCh than controls (GFP^+^). (**b**) Magnification of the white box in **a**. (**c**) Quantification of the apical and basal levels of Sqh-mCh in control and *flw* mutant FCs. The statistical significance of differences was assessed with a *t*-test, *** *P* value<0.0001. All errors bars indicate s.d. (**d**,**l**) Surface view of control S8 (**d**–**f**), mosaic S8 (**g**–**i**) and control S10 (**j**–**l**) egg chambers expressing Sqh-mCherry, stained for anti-GFP (green) and Rhodamine-Phalloidin to detect F-actin (red). (**g**–**i**) The actomyosin fibers of *flw* mutant FCs exhibit higher levels of F-actin and myosin compared with controls (**d**–**f**). Scale bar, 10 μm. Mean of *n*>42 FCs, assessed over five independent experiments.

To address the role of *flw* in actomyosin contractility regulation in FCs, we analysed myosin localization (by means of Sqh-GFP accumulation) in egg chambers carrying FCs homozygous for the null allele *flw*^*FP41*^ (ref. 15; 10 independent egg chambers were scored). We found that while basal Sqh-GFP levels increased nearly twofold (46%) in S6 *flw* mutant FCs (*n*=50) compared with controls (*n*=52), apical levels only increased by 18% (Fig. 2a–c). As the catalytic subunit of phosphatases can be highly pleiotropic, to ensure that Flw was controlling myosin dynamics directly, as opposed to indirectly, we tested whether elimination of the MLCP-binding subunit Mbs would also lead to an increase in myosin activity. To test this, we measured the levels of activated Sqh in FCs mutant for *flw* or *Mbs,* using an antibody that specifically recognizes Sqh when it is phosphorylated at the activating Ser-21 (pSqh)^16^. We found that both *flw* and *Mbs* mutant FCs displayed higher basal levels of pSqh compared with control (Supplementary Fig. 1). Next, we characterized the actomyosin fibres in both control and *flw* mutant FCs, identified by co-staining of F-actin and Sqh-GFP. We found higher levels of both actin and myosin in the fibres of S8 *flw* mutant FCs (*n*=48) compared with controls (*n*=42; Fig. 2d–i, >12 different egg chambers were analysed), which resemble those found in wild-type FCs at later stages^12^ (Fig. 2j–l, *n*=46). This similarity between S8 *flw* mutants and S9–S10 wild-type FCs unveils a role for *flw* in the control of the timing and amount of F-actin and myosin recruited basally. Hence, our results suggest that variations in the sub-cellular distribution of Flw might act as a switch between early- and late-type of basal actomyosin structures.

**Figure 3:**
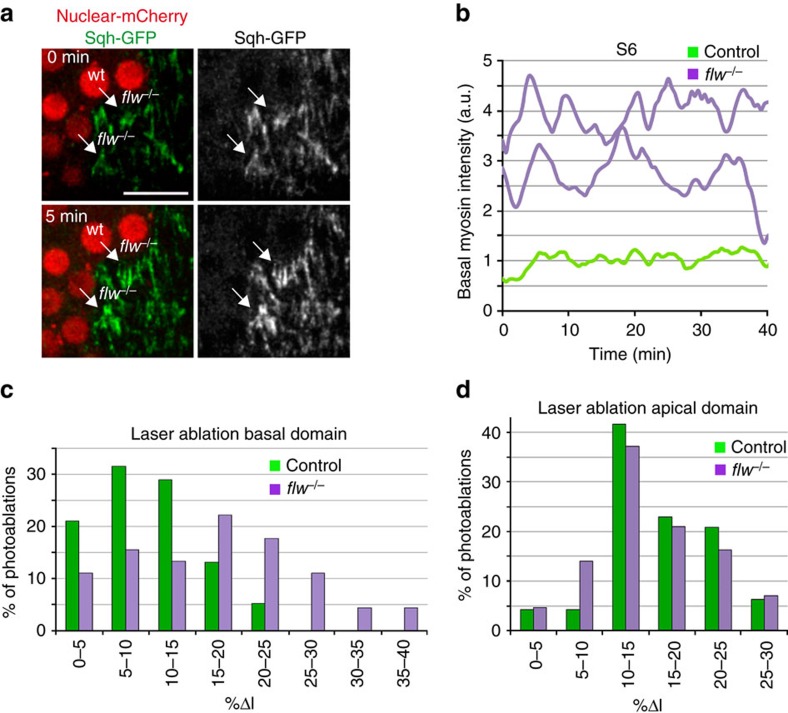
Loss of *flw* results in both precocious basal myosin oscillations and increased basal contractility. (**a**) Confocal images taken with a 5-min difference of a live S6 mosaic egg chamber expressing Sqh-GFP (green) and carrying *flw* mutant clones identified by the absence of nuclear mCherry (red). While the levels of Sqh-GFP in *flw* mutant FCs are high and oscillate (arrows), control cells show low and constant Sqh-GFP amounts. (**b**) Quantification of the dynamic changes of Sqh-GFP in one of the above control cells (light green line) and two *flw* mutants (purple line). (**c**,**d**) Quantification of cell elongation after photoablation at the basal (**c**) and apical (**d**) domains of control (*n*=52) and *flw* (*n*=56) mutant FCs in six independently cultured egg chambers. %Δl=100x(*L−l*)/*l* where *l* is the original distance between the cell vertices associated to the ablated edge and *L* is the distance 10 sec. after photoablation. Scale bar: 10 μm. Mean of *n*>30 FCs, assessed over 9 independent experiments.

A characteristic feature of the basal contractile arrays formed in S9–S10 wild-type FCs is the periodic changes in myosin concentration^8^. To test if the elevated levels of basal myosin found in early-stage *flw* mutant FCs underwent a similar oscillatory behaviour, we performed live imaging of mosaic S6 egg chambers expressing Sqh-GFP and containing *flw* mutant FCs (nine egg chambers cultured independently on different days were analysed). We found that basal myosin in mutant cells (*n*=30) underwent periodic fluctuations (Fig. 3a,b; Supplementary Movie 1). However, in contrast to the marked and periodic changes in basal myosin levels found in S9–S10 control FCs^8^ (*n*=34, Supplementary Movie 2), the oscillation period in S6 *flw* mutant FCs showed a high degree of variability (Fig. 3b), suggesting that *flw* regulated not only the initiation of myosin oscillation but also its periodicity.

Laser ablation of cell–cell boundaries has emerged as a powerful tool to measure actomyosin contractility-based intracellular tensions^17^. Thus, to test whether the basal actomyosin structures found in S8 mutant cells changed their contractility state, we used a UV laser beam to sever plasma membranes and the cortical cytoskeleton in both control and *flw* mutant FCs at S8. Cell boundaries were ablated just below the basal plasma membrane and the behaviour of cell membranes, visualized with Spider-GFP (a live membrane marker), was monitored 10** **s after ablation. As a consequence of the cut, cortical tension relaxed and the distance between the cell vertices at both sides of the cut increased. As velocity of retraction is affected by cytoplasmic viscosity^18^, we assumed viscosity in *flw* and wild-type FCs to be comparable. To minimize possible effects due to the anisotropic distribution of forces in the follicular epithelium at this stage, cuts were all made parallel to the anterior–posterior axis and in the central region of eight independently cultured egg chambers. In addition, only vertices initially separated by similar distances, between 4.5 and 5.5 μm, were considered. We found that the percentage of distance increase (%Δl, calculated as 100x(*L−l*)/*l*, where *L* and *l* are the final and original distances, respectively) of *flw* mutant FCs was significantly higher than that observed in controls (Fig. 3c). Thus, only 18% of control FCs (*n*=52) showed a %Δl above 15, in contrast to the 62% found in *flw* mutant FCs (*n*=56; Fig. 3c). The increase in contractility was specific of the basal side, as there was no difference between control and *flw* mutant FCs when the laser ablation was performed apically (Fig. 3d, *n*=48). Altogether, these results strongly suggest that elimination of *flw* in FCs leads to an increase in their basal actomyosin-dependent contractility.

### Flw regulates oscillatory dynamics of basal myosin

**Figure 4:**
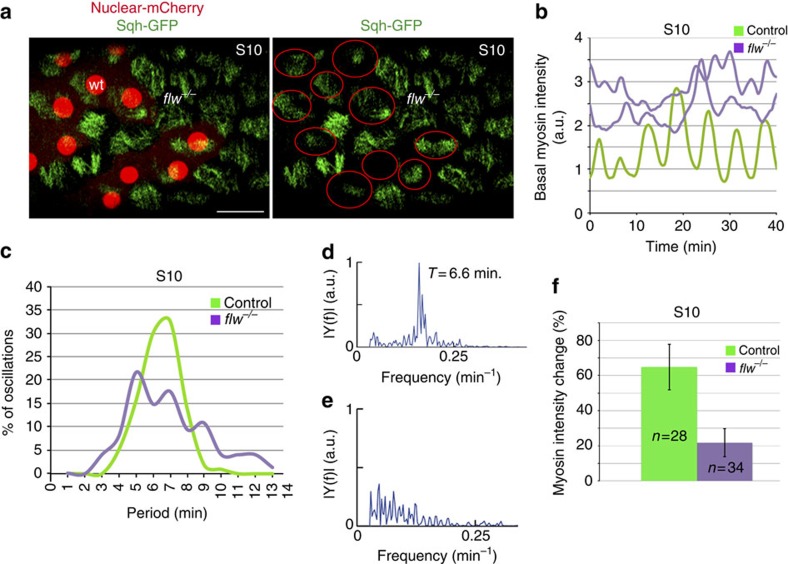
Flw regulates the periodicity of the basal myosin oscillations. (**a**) Confocal image of a live S10 mosaic egg chamber expressing Sqh-GFP (green) and carrying *flw* mutant clones identified by the absence of nuclear mCherry (red). Red circles denote control FCs. (**b**) Quantification of the dynamic changes of basal Sqh-GFP in one S10 control FC (light green) and two *flw* mutant FCs of the same stage (purple). (**c**) Distribution of the frequency of basal Sqh-GFP oscillatory periods observed in control and mutant FCs. (**d**,**e**) Fourier transform of the autocorrelation function of temporal sequences of basal myosin intensity for control (**d**) and *flw* mutant (**e**) FCs. (**f**) Quantification of basal myosin intensity changes in S10 control and *flw* mutant FCs. Scale bar, 10 μm. Mean of *n*>27 FCs, assessed over nine independent experiments. All errors bars indicate s.d.

Our *in vivo* analysis of S6 *flw* mutant FCs suggested that, in addition to its role in regulating the onset of contractility, *flw* could also control the periodicity of basal myosin oscillations. To test this, we analysed mosaic follicles expressing Sqh-GFP and bearing *flw* mutant FCs (at least eight egg chambers cultured independently in different days were analysed). We decided to study S9–S10 follicles, since at these stages egg chamber rotation has stopped and periodic basal myosin oscillations of both control and mutant cells can be monitored in greater detail^8^. We found that, as it was the case for S6 FCs, S10 *flw* mutant FCs (*n*=34) showed higher levels of basal myosin than controls (*n*=28; Fig. 4a). To gain further insight into the dynamics of basal myosin accumulation we performed time-lapse imaging on living follicles from eight independently cultured egg chambers and found that, even though both control (*n*=27) and *flw* (*n*=32) mutant FCs showed temporal variations in basal myosin amounts, their behaviour was markedly different (Supplementary Movie 3, Fig. 4b,c). First, to quantify myosin dynamics, we developed a Matlab script to measure pulsation periodicity and observed that the preferential pulsation frequency of control FCs was – in agreement with previous results^8^—a 6.6-min period (Fig. 4d, *n*=27). However, mutant FCs did not exhibit a clear frequency peak; rather, they display stochastic pulsations of myosin accumulation (Fig. 4e, *n*=32). Second, the amplitude of the variations in the amount of basal myosin was considerably lower in *flw* mutant FCs (∼20%, *n*=34) compared with controls (∼65%, *n*=28; Supplementary Movie 3 and Fig. 4f). Altogether these results showed that *flw* is required to regulate the periodicity and the intensity of the fluctuations in basal myosin accumulation.

### Basal F-actin and myosin levels show similar dynamics

**Figure 5:**
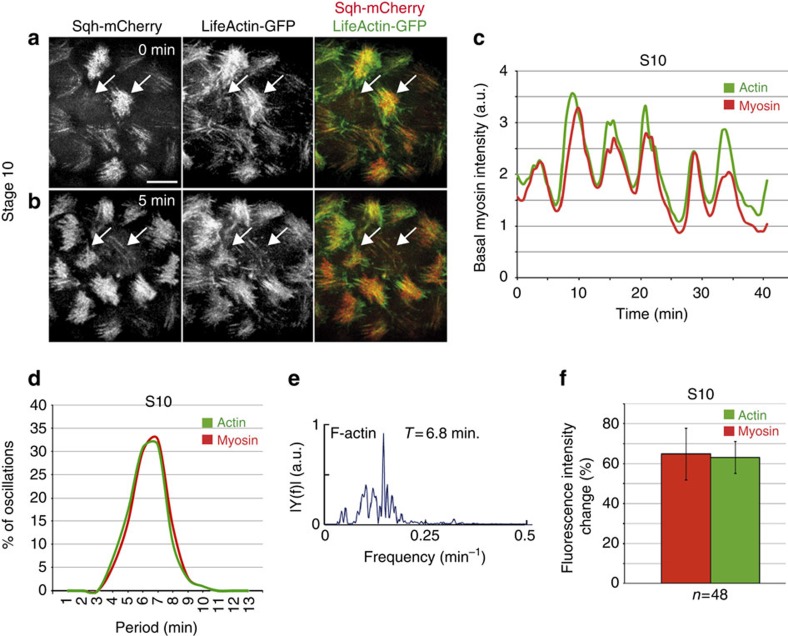
Basal Lifeact-GFP oscillates with the same periodicity and amplitude as myosin. (**a**,**b**) Confocal images of live S10 egg chamber expressing Sqh-mCherry (red) and Lifeact-GFP (green) taken with a 5-min interval. During this time, basal F-actin and myosin intensity changes in two FCs (arrows). (**c**) Quantification of the dynamic changes of basal myosin (Sqh-mCherry) and F-actin (Lifeact-GFP) in one oscillating FC. (**d**) Distribution of the frequency of basal myosin (Sqh-mCherry) and F-actin (Lifeact-GFP) oscillatory periods. (**e**) Fourier transform of the autocorrelation function of temporal sequences of basal F-actin intensity for control FCs. (**f**) Quantification of Sqh-mCherry and Lifeact-GFP intensity changes in S10 FCs. Scale bar, 10 μm. Mean of *n*>27 FCs, assessed over nine independent experiments. All errors bars indicate s.d.

Previous work in *Drosophila* embryos has shown that pulsed contractions of an actomyosin network drive apical cell surface area reduction during gastrulation and dorsal closure^11^,^19^. In these morphogenetic events, both myosin and actin filaments show a pulsatile behaviour. In contrast, it has been reported that while basal myosin concentrations oscillate ∼70% in FCs, the basal F-actin levels changed only ∼20% over time, as visualized by Moesin-GFP. This led to the suggestion that the periodic changes in basal myosin levels were not produced by dynamic alterations in F-actin^8^. However, we had noticed that basal F-actin accumulation at S9–S10 showed a high degree of variability in intensity in fixed samples (Fig. 2j). This finding prompted us to perform time-lapse imaging of S9–S10 egg chambers expressing both Sqh-mCherry and any of three markers often used to monitor F-actin dynamics: Lifeact-GFP, Utrophin-GFP or Moesin-GFP^20^,^21^,^22^ to record simultaneously dynamic changes in myosin and F-actin (at least nine egg chambers cultured independently in different days were analysed for all experimental conditions). Corroborating our findings in fixed samples, we observed that F-actin levels oscillated with the same periodicity and amplitude as myosin with the three markers utilized, Lifeact-GFP, Utrophin-GFP and Moesin-GFP (Supplementary Movie 4–8; Fig. 5; Supplementary Fig. 2, *n*>48). The discrepancy between our results and those previously published^8^ could arise from the differences in the mounting conditions (Methods section). Taken altogether, our results demonstrate that basal F-actin levels oscillate similarly to myosin in FCs. Furthermore, simultaneous quantification of the intensity of Sqh-mCherry and Lifeact-GFP over time revealed that, while both proteins oscillated with similar periodicity and amplitude, in ∼54% of the cases analysed (*n*=19), actin accumulation preceded that of myosin by ∼42** **s. The other 46% of cases showed simultaneous pulsation of F-actin and myosin levels.

Altogether, these results strongly suggest that assembly and disassembly of basal F-actin and myosin fibres are coordinated during egg chamber elongation (S9–S10) and that Flw activity is required to coordinate these actomyosin dynamics. In this scenario, we would expect basal F-actin levels to behave similarly to myosin in *flw* mutant FCs. Indeed, our analysis of basal F-actin dynamics in S6 *flw* mutant FCs showed, as it was the case for myosin levels, premature oscillation (Supplementary Fig. 3, Supplementary Movie 9), thus supporting our hypothesis.

Next, we decided to incorporate the above observations into an in silico model to investigate possible mechanisms responsible for F-actin and myosin periodic pulsations.

### A cell-autonomous model of basal actomyosin oscillations

**Figure 6:**
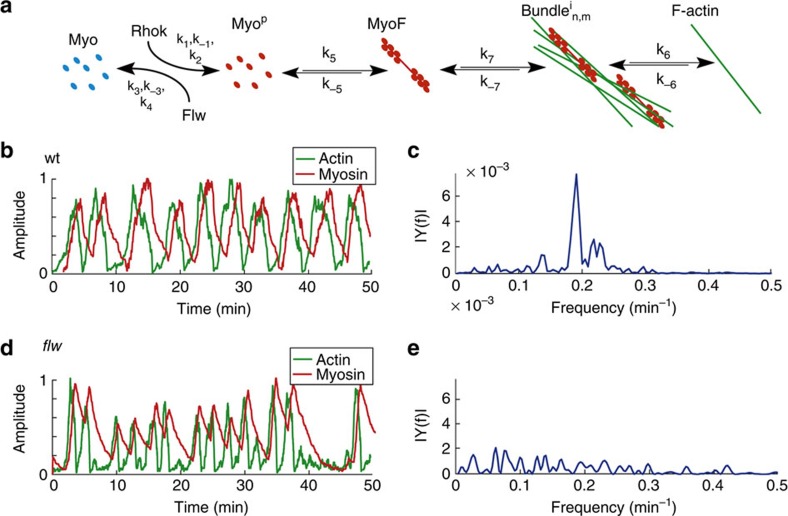
Simulations of basal actomyosin oscillations in wild-type and *flw* mutant FCs. (**a**) A simplified model for actomyosin interactions. See Online Methods section and Supplementary Information for details. (**b**,**d**) Simulations of basal actomyosin oscillations in (**b**) wild-type, and (**d**) *flw* mutant FCs. (**c**,**e**) Fourier transform of the autocorrelation function of temporal sequences of basal myosin intensity for wild-type (**c**), and *flw* mutant (**e**) FCs.

While several mathematical models have been proposed to explain actomyosin oscillations exhibited by apically contracting cells (reviewed in refs 23, 24), very few theoretical studies have addressed the oscillations that occur at the basal surface of cells^9^. In our model, we incorporated our observations of control and mutant cells to two general properties of the actomyosin network: cooperative actin bundling^25^ and disassembly of actin bundles due to myosin-induced tension^26^ (Fig. 6a). The interplay between these two reactions is sufficient to produce autonomous oscillations that reproduce the features of the basal oscillations observed during egg chamber elongation, in both wild type and mutant conditions. This minimal but robust model (Online Methods section and Supplementary Material for details) was able to make the following predictions: (1) that actomyosin filaments in FCs oscillate with an average period of 6–8 min and oscillations in myosin and F-actin intensities correlate (Fig. 6b,c), (2) that actin accumulation precedes that of myosin by ∼60** **s (Fig. 6b) and finally, that reducing Flw total concentration by three orders of magnitude (equivalent to *flw* loss of function; Supplementary Table 1, [*Flw*^*T*^]_lof_ compared with [*Flw*^*T*^]_S9–S10_) resulted in a transition to stochastic bursting in myosin and actin accumulation (Fig. 6d,e). Our model recapitulates basal actomyosin dynamics at the cellular level and suggests, in contrast to previously proposed models^9^, a cell-autonomous mechanism for the emergence of basal oscillations of both myosin and F-actin *in vivo*.

**Figure 7:**
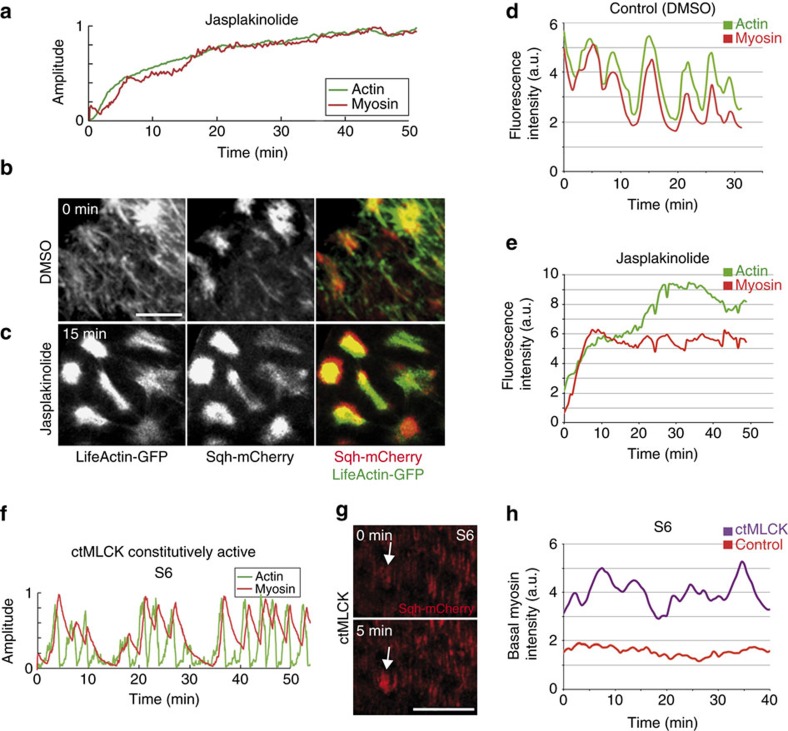
Basal actomyosin dynamics in jasplakinolide-treated FCs and FCs overexpressing ctMLCK. (**a**) Simulation of basal actomyosin oscillations in jasplakinolide-treated FCs. (**b**) Life confocal image of the basal side of S10 FCs expressing Lifeact-GFP and Sqh-mCherry, 15 min after incubation with DMSO-containing culture medium. (**c**) Live imaging of the same egg chamber shown in (**b**) 15 min. after jasplakinolide addition. (**d**,**e**) Quantification of the dynamic changes of basal myosin (Sqh-mCherry, red) and F-actin (Lifeact-GFP, green) in one FC treated as above. (**f**) Simulation of basal actomyosin oscillations in FCs expressing ctMLCK. (**g**) Live confocal images of the basal side of S10 FCs expressing Sqh-mCherry and ctMLCK. The arrow points to a FC that has changed its basal level of Sqh-mCherry in 5 min. (**h**) Quantification of the dynamic changes of basal myosin (Sqh-mCherry) in control (red line) and ctMLCK (purple line) expressing FCs. Scale bar, 10 μm. Mean of *n*>32 FCs, assessed over eight independent experiments.

We next decided to challenge the model by simulating two experimental situations and then testing the model predictions in perturbation experiments, using pharmacological or molecular manipulations. First, our model predicted that preventing F-actin oscillations would block myosin pulsations. To simulate this, we stabilized F-actin by reducing *k*_*−6*_ in equation (6) (see Modelling actomyosin basal oscillations in Supplementary Information). In our simulations, this led to a noticeable increase in F-actin and myosin accumulation and disruption of the oscillatory dynamics of both actin and myosin (Fig. 7a). To test this experimentally, we undertook a pharmacological approach and manipulated actin filaments using the F-actin-stabilizing drug jasplakinolide^27^. In contrast to control samples cultured in the solvent (dimethylsulphoxide (DMSO); Fig. 7b,d and Supplementary Movie 10, *n*=32), the addition of jasplakinolide increased the density of basal F-actin filaments, preventing the normal fluctuations of the Lifeact-GFP marker (Fig. 7c,e and Supplementary Movie 11, *n*=38). As predicted by the model, myosin oscillations were also blocked (Fig. 7c,e and Supplementary Movie 11). Second, the model also predicted that ectopic activation of the myosin light-chain kinase (MLCK) would lead to premature pulsation. We simulated this by increasing *k*_*2*_ in equation (1) (see Modelling actomyosin basal oscillations in Supplementary Information), which led to precocious, asynchronous oscillations at S6 (Fig. 7f), similar to what happens when eliminating *flw* function (Fig. 6d). We next tested this experimentally by co-expressing a constitutive form of MLCK (ref. 28; ctMLCK) and Sqh-mCherry in FCs. As anticipated by the model, basal myosin oscillations were observed in S6 FCs expressing ctMLCK (Fig. 7g,h and Supplementary Movie 12). In summary, our model makes robust predictions, as it reproduces the features of actomyosin oscillations in wild type as well as on experimental perturbations.

## Discussion

In recent years, actomyosin-dependent contractile forces have emerged as key regulators of the oscillatory behaviour underlying morphogenesis (reviewed in refs 29, 30). However, the mechanisms controlling the emergence and periodicity of actomyosin oscillations remain unclear. In this work, we have addressed these two questions. On one hand, we have identified the *Drosophila* MLCP-component Flw as a novel, key regulator of both the onset and periodicity of the basal actomyosin oscillations that occur in FCs. On the other, we have shown that, in contrast to what has been previously published^8^ and similar to what happens during apical constriction events^31^, basal F-actin levels oscillate with a similar amplitude and periodicity to that of myosin. This has led us to propose a simple model for the emergence of actomyosin oscillations in which the combination of cooperative binding of actin filaments in conjunction with actin filament dissociation from the bundle, due to myosin-induced tension, is sufficient to generate cell-autonomous oscillations in myosin and F-actin content.

In *Drosophila*, Flw was shown to prevent over-contraction of larval muscles, nurse cells' ring canals and imaginal disc epithelium^32^,^33^,^34^. Similarly, we find that loss of *flw* increases basal cell contractility. Our observations that basal accumulation of Flw in FCs decreases when contractility starts and that *flw* elimination causes premature actomyosin oscillations unravel a novel function for *flw* as an intracellular timer regulating the onset of basal actomyosin oscillations. However, a number of questions still remain unanswered. For instance, how is Flw localization regulated during egg chamber development? Recently, a model has been proposed in which the Rho-RhoK pathway is activated in FCs due to pressure exerted by the underlying growing germline cells^9^. As RhoK inactivates by phosphorylation the myosin-binding subunit of MLCP (ref. 35), which regulates the affinity of PP1 for phosphorylated myosin^36^, stimulation of Rho-Kinase signalling in FCs might serve two purposes, activation of myosin and attenuation of Flw function, thus ensuring that the right amount of activated myosin is reached. Our results also show that the reduction of Flw levels at the onset of FC contractility is specific for the basal side of the cells, as apical amounts remain constant and apical tension does not change in the absence of *flw*. We would like to propose that the spatial regulation of Flw localization and/or activity could depend on a local activation of specific kinases on the apical versus the basal regions of FCs due to different upstream stimuli. In fact, experiments in serum-starved 3T3 cells have shown that two different MLCKs, RhoK and MLCK, function at different cellular locations^37^. In the future, it will be interesting to analyse the distribution and function of the different MRLC kinases during FC basal oscillations. In addition, forthcoming studies could also be directed to determine whether local recruitment of Flw is a widespread mechanism to regulate actomyosin oscillations in other cell types and in other locations within the cell, such as the apical oscillations underlying *Drosophila* mesoderm invagination or dorsal closure (reviewed in refs 2, 30). This would lead to a better understanding of how organization and dynamics of the different components of the contractile machinery leads to distinct mechanical properties of tissues that regulate organ and tissue shape.

Biochemical analysis and studies in intact smooth muscle cells have implicated MLCP in the modulation of MRLC phosphorylated levels and hence cell contraction. However, insights into a similar role of MLCP *in vivo* are very limited and mainly derived from studies in smooth muscle cells. Research in zebrafish showed that myosin phosphatase was required to regulate hindbrain morphogenesis by apical modulation of myosin function^38^. Here, we show that loss of *flw* from FCs leads to an increase in the basal levels of MRLC and to changes in the basal actomyosin oscillatory behaviour of these cells. From these results, we propose that while the MLCP-component Flw is not an essential effector of actomyosin-dependent oscillations, it serves a modulatory role. Interestingly, similar to what we describe here for Flw, smooth muscle specific knockout of the myosin-binding subunit of the MLCP (MYPT1) in mice leads to changes in the contractile properties of gut phasic and tonic vascular smooth muscles^39^,^40^. Thus, as it is the case for Flw, MYPT1 is necessary for proper contractility in smooth muscle cells, albeit not essential for muscle contraction. These results strongly suggest that the role of MLCP in the modulation of cell contractility is conserved between species and across different cell types. An imbalance of RhoK and MLCP in smooth muscle cells, resulting in sustained phosphorylation of MRLC, contributes to the pathogenesis of many vascular diseases, such as vasospasm, hypertension and asthma, and intestinal motility dysfunction^40^,^41^,^42^,^43^. Thus, deciphering the specific role(s) of MLCP will help us not only to further understand MRLC phosphorylation and myosin activation but also to design new drugs to treat diseases caused by abnormal smooth muscle contraction. The similarities between the function of MLCP in FCs and smooth muscle cells makes *Drosophila* FCs an ideal model system to investigate the regulation of its activity and its role in regulating MLC activity and cell contraction. Finally, it has been shown that the catalytic subunit of PP1 phosphatases are very promiscuous and besides the myosin regulatory subunit they can bind other proteins, including actin-binding proteins, such as neurabin I (NrbI)^44^ and spinophilin^45^. Furthermore, experiments in neurons have shown that although PP1c binding to NrbI does not dictate its phosphorylation state, it is required for the ability of NrbI to bind F-actin and to reorganize the actin cytoskeleton by disassembling stress fibres^46^. In this context, in addition to its role on myosin activity, Flw could also contribute to the regulation of actomyosin dynamics by directly controlling the activity of actin-binding proteins. In the future, it will be interesting to determine whether proteins capable of interacting with Flw are expressed in FCs and if this were the case analyse whether they have any role on regulating actomyosin dynamics.

**Figure 8:**
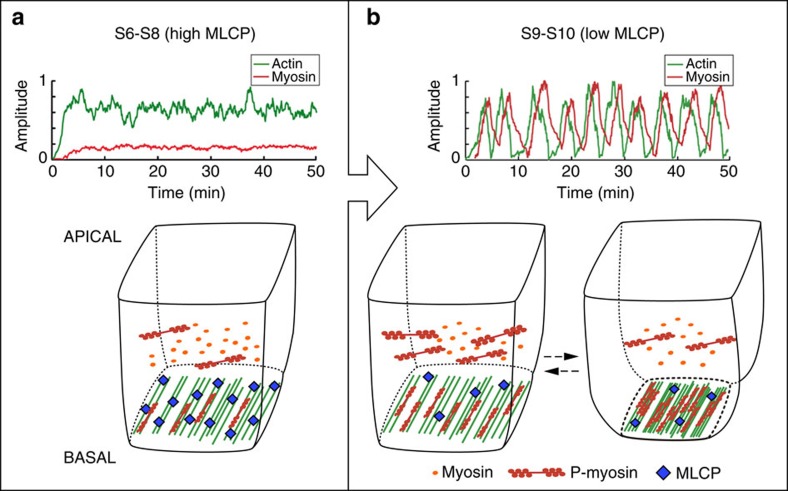
A cell-autonomous model for the initiation and maintenance of periodic basal actomyosin oscillations in FCs. Simulations of basal F-actin and myosin behaviour and the consequences for cell contraction at (**a**) high concentrations of Flw (2.7 μM, which resemble the conditions of S6–S8 egg chambers) and (**b**) low concentrations (0.9 μM; S9–S10 egg chambers). In both situations the amounts of myosin were set at 0.4 μM. At S9–S10, the oscillations of actomyosin levels result in the pulsed contraction of basal cell surfaces.

It has been known for a number of years that the contractile apparatus of certain muscle types, such as striated skeletal and cardiac muscle fibres, exhibit spontaneous oscillations in culture^47^. In addition, *in vitro* studies have shown that a minimal actomyosin system under elastic loading can oscillate spontaneously without the need of the regulatory proteins present *in vivo*^48^. Here, we propose that actomyosin oscillations governing egg chamber elongation can in principle arise generically from the dynamic properties of motor assemblies. This is in contrast to previous models for actomyosin oscillations occurring at the apical side of cells during *Drosophila* dorsal closure, which involve the need of complex protein turnover dynamics^49^,^50^ or the effect of external oscillatory signals^51^. Our model also differs from a recent proposition for the basal myosin fluctuations happening in FCs in which oscillations emerge without the need of periodic actin basal accumulation and assuming a nonlinear Rho activation coupled to mechanical tension^9^. We suggest that basal actomyosin oscillations occur above a threshold of active myosin and can run constitutively, independently of external forces and simply due to the interplay between cooperative actin aggregation and disassembly of the network (Fig. 8). Importantly for the initiation of actomyosin oscillations, our results point to a critical role for MLCP in the regulation of activated myosin levels. In our model, a cell with a given concentration of myosin (which can be found in an active or inactive state and that we estimate in 0.4 μM (Supplementary Table 1)) elicits different responses depending on the levels of the regulatory phosphatase. Thus, our simulations show that high levels of basal Flw (such as the ones found in FCs prior to S9, estimated as 2.7 μM) prevent oscillations, while the decrease in Flw amounts at S9 (0.9 μM in our *in silico* simulations) allows FCs to reach enough phosphorylated myosin concentrations as to initiate pulsation (Fig. 8). Finally, since our simulations define periodic F-actin and myosin fluctuations for concentrations of Flw between 0.2 and 1.25 μM, the oscillatory behaviour of the basal actomyosin cytoskeleton of S9–S10 FCs is significantly robust. It will be interesting to determine whether our model could also account for the actomyosin oscillations taking place on the apical side of the cells during morphogenesis. Understanding the commonalities and variations between apical and basal oscillations promises to provide further insight into the mechanisms underlying tissue morphogenesis and to improve tissue engineering protocols.

## Methods

### *Drosophila* stocks and genetics

The following fly stocks were used: *flw-YFP-159*, a fusion protein-trap line in which a YFP-coding P-element is inserted in the *flw* gene^10^; *flw*^*FP41*^*FRT19A/*FMZ (ref. 15); Sqh-GFP (ref. 52) and Sqh-mCherry (refs 11, 52); UASp-UtrGFP (ref. 53), *MbsT666FRT80/TM6B* (ref. 54), UAS-Lifeact-GFP, UAS-Moesin-GFP, *nlsGFP FRT19A*, *w[1118]; Ubi-mRFP.nlsFRT80B* and Spider-GFP from the Bloomington *Drosophila* Stock Centre, UAS-ctMLCK (ref. 28) and the follicle stem cell driver *traffic jam*-Gal4 (tj-Gal4 (ref. 55)). The *e22c-gal4* driver is expressed in the follicle stem cells in the germarium and was therefore used in combination with *UAS-flp* to generate *flw* and *Mbs* FC clones. To analyse myosin and F-actin dynamics in *flw* mutant clones, *flw*^*FP41*^*FRT19A/*FMZ; Sqh-GFP or *flw*^*FP41*^*FRT19A/*FMZ; Lifeact-GFP females were crossed to *nlsGFP FRT19A; e22c-gal4 UAS-flp/CyO* and *hsflpRFPFRT19A* males, respectively. To generate *Mbs* mutant clones, *MbsT666FRT80/TM6B* females were crossed to *e22c-gal4 UAS-flp/CyO;Ubi-mRFP.nlsFRT80B.* To analyse actin and myosin dynamics simultaneously, we generated females carrying *Sqh-mCherry, UAS-Lifeact-GFP* and *tj-Gal4* transgenes. Flies were grown at 25 °C and yeasted for 2 days before dissection.

### Time-lapse image acquisition

For live imaging 1–2-day-old females were fattened on yeast for 48–96 h before dissection. Culture conditions and time-lapse microscopy was done using a modified version of the protocol described in ref. 56. *Drosophila* ovaries were dissected and mounted using the same medium^56^, Schneider's insect medium (GIBCO-BRL) supplemented with 10% foetal bovine serum (F3018; Sigma), 0.63 penicillin/streptomycin (GIBCO) and 0.20 mg ml^−1^ insulin (I5500; Sigma), with a final pH adjusted to 6.9. However, egg chambers were not mounted on a 50-mm Petriperm plate (Greiner Bio) and covered with a 22-mm coverslip as in ref. 56. Instead, egg chambers were mounted on a 35-mm glass bottom poly-D-lysine-coated dish (MatTek Corporation) without coverslip. In addition, a roll of wet tissue paper was placed surrounding the glass bottom to preserve a certain degree of humidity around the culturing egg chambers. Movies were acquired on a Leica SP5 MP-AOBS confocal microscope equipped with a 40 × 1, 3 PL APO oil objective. *Z*-stacks with 11–12 slices (0.42 μm interval) were taken to capture the entire basal myosin and actin filaments. Frames were taken every 30** **s.

Laser ablation experiments were performed on an Olympus IX-81 spinning disc microscope equipped with a Yokogawa CSU-X1 scanning head, a × 60 (1.35 NA) UplanSApo oil objective, a CoolSnap HQ2 camera and a 355-nm iPulse laser (Roper Scientific) with a pulse energy of 2.5 J, 6 kHz, 400 ps. Dwell time was 100 msec. Images were taken immediately before and 10** **s after laser pulse.

### Image processing and data analysis

Quantification of the degree of co-localization between different markers was performed using Fiji and the PCC in maximum projections images. The analysis was performed in individual FCs. Furthermore, as, Flw-YFP signal was only detected at the tips of the F-actin and Sqh-mCherry bundles in S6 egg chambers, in this case the analysis was done specifically in this region, to avoid under-representing the degree of correlation between the different markers.

For quantification of basal myosin and actin dynamics over time, maximal projections of confocal stacks were produced to account for egg chamber curvature. Integrated intensity of myosin (red) and actin (green) were quantified for manually selected regions using ImageJ software. The background value taken from cell-free regions was subtracted from all data series. Quantification of inter-vertex distances in the photoablation experiments was performed manually using ImageJ software.

The distribution of oscillation periods was obtained by measuring the intervals between each pair of two adjacent peaks. Myosin intensity changes in both wild-type and MLCP mutant cells were obtained by averaging the difference between the maximum and the minimum fluorescence intensity for each oscillation. To calculate the period of myosin and actin oscillation, a Matlab script was developed to measure the power spectrum density of the signal using one-dimensional Fourier transform of the autocorrelation function (code available on request).

Statistical analysis of significant differences between control and experimental samples was done using Student's *t*-test.

### Drug treatment

Ovarioles were dissected in live-imaging medium and mounted for imaging as described above in the time-lapse image acquisition section. S9–S10 egg chambers were first imaged for 30 min. On addition of DMSO to control follicles (at a final concentration of 0.6%) or Jasplakinolide (dissolved in DMSO and at a final concentration of 6 μM, Sigma Aldrich), the same egg chambers were imaged for another 40 min.

### Immunohistochemistry

For fixed samples, ovaries were dissected at room temperature in Schneider's medium (Sigma Aldrich) to preserve cytoskeletal structures. Fixation was performed incubating egg chambers for 20 min with 4% paraformaldehyde in PBT (phosphate-buffered saline+0.1% Tween 20). For actin labelling, fixed ovaries were incubated with Rhodamine-Phalloidin (Molecular Probes, 1:200) for 30 min. The DNA dye TO-PRO-3 (Molecular Probes) was used at 1:1.000 in PBT. Samples were mounted in Vectashield (Vector Laboratories). Images were acquired on a Leica SPE confocal microscope equipped with a × 40 (1.15 NA) ACS-APO oil objective.

## Additional information

**How to cite this article:** Valencia-Expósito, A. *et al.* Myosin light-chain phosphatase regulates basal actomyosin oscillations during morphogenesis. *Nat. Commun.* 7:10746 doi: 10.1038/ncomms10746 (2016).

## Supplementary Material

Supplementary Information Supplementary Table 1, Supplementary Notes 1-3 and Supplementary References

Supplementary Movie 1 Basal myosin oscillations in live S6 flw mutant FCs Time-lapse movie of a S6 mosaic egg chamber expressing Sqh-GFP (green) and carrying flw mutant clones identified by the absence of nuclear mCherry (red). Focus is on the basal surface. In contrast to control FCs, myosin intensity oscillates in flw mutant FCs. Note the characteristic rotation of S6 egg chambers.

Supplementary Movie 2 Basal myosin oscillations in live S10 control FCs. Time-lapse movie of a S10 control egg chamber expressing Sqh-mCherry (red). Note the periodic pulsation in myosin intensity typical of basal FC surfaces.

Supplementary Movie 3 Basal myosin oscillations in live S10 flw mutant FCs Time-lapse movie of a S10 mosaic egg chambers expressing Sqh-GFP (green) and carrying flw mutant clones identified by the absence of nuclear mCherry (red). Focus is on the basal surface. Note that the changes in myosin intensity in flw mutant FCs are much reduced than in controls.

Supplementary Movie 4 Basal myosin and F-actin oscillations in live S10 control FCs. Time-lapse movie of a S10 control egg chamber expressing Lifeact-GFP (green) and Sqh-mCherry (red). Focus is on the basal surface. Oscillations in F-actin intensity occur with the same amplitude and periodicity than those of myosin.

Supplementary Movie 5 Basal myosin and F-actin oscillations in live S10 control FCs. Time-lapse movie of a S10 control egg chamber expressing Lifeact-GFP (green) and Sqh-mCherry (red). Focus is on the basal surface. Oscillations in F-actin intensity occur with the same amplitude and periodicity than those of myosin. Supplementary Movie 5 corresponds to the Lifeact-GFP and Sqh-mCherry

Supplementary Movie 6 Basal myosin and F-actin oscillations in live S10 control FCs. Time-lapse movie of a S10 control egg chamber expressing Lifeact-GFP (green) and Sqh-mCherry (red). Focus is on the basal surface. Oscillations in F-actin intensity occur with the same amplitude and periodicity than those of myosin. Supplementary Movie 6 corresponds to the Lifeact-GFP and Sqh-mCherry channels.

Supplementary Movie 7 Utrophin-GFP reports basal F-actin oscillations in live S10 control FCs. Time-lapse movie of a S10 control egg chamber expressing Utrophin-GFP (green). Imaging of the basal side of FCs reveals dynamic oscillations in F-actin similar to those observed using Lifeact-GFP.

Supplementary Movie 8 Moesin-GFP reports basal F-actin oscillations in live S10 control FCs. Time-lapse movie of a S10 control egg chamber expressing Moesin-GFP (Moe-GFP, green). Imaging of the basal side of FCs reveals dynamic oscillations in F-actin similar to those observed when using Lifeact-GFP.

Supplementary Movie 9 Basal F-actin oscillations in live S6 flw mutant FCs. Time-lapse movie of a S6 mosaic egg chamber expressing Lifeact-GFP (green) carrying flw mutant clones identified by the absence of nuclear RFP (red). Focus is on the basal surface. In contrast to control FCs, F-actin intensity oscillates in flw mutant FCs. Note the characteristic rotation of S6 egg chambers.

Supplementary Movie 10 Effects of jasplakinolide on F-actin and myosin accumulation and dynamics. Time-lapse movies of a S10 control egg chamber expressing Lifeact-GFP (green) and Sqh-mCherry (red) and cultured in medium containing either the vehicle alone (DMSO, Movie S7) or DMSO + jasplakinolide (Movie S8). Imaging of the basal side of FCs reveals the drastic effects of the drug on F-actin and myosin accumulation and dynamics.

Supplementary Movie 11 Effects of jasplakinolide on F-actin and myosin accumulation and dynamics. Time-lapse movies of a S10 control egg chamber expressing Lifeact-GFP (green) and Sqh-mCherry (red) and cultured in medium containing either the vehicle alone (DMSO, Movie S7) or DMSO + jasplakinolide (Movie S8). Imaging of the basal side of FCs reveals the drastic effects of the drug on F-actin and myosin accumulation and dynamics.

Supplementary Movie 12 Basal myosin oscillations in live S6 FCs expressing ctMLCK. Time-lapse movie of a S6 egg chamber expressing Sqh-mCherry (red) and ctMLCK. Focus is on the basal surface. In contrast to control FCs, myosin intensity oscillates in FCs expressing ctMLCK. Note the characteristic rotation of S6 egg chambers.
