# Supplementary material for: Myosin light-chain phosphatase regulates basal actomyosin oscillations during morphogenesis
Source: Nat Commun. 2016 Feb 18;7:10746. doi: 10.1038/ncomms10746 (PMC4759631; doi:10.1038/ncomms10746)
Supplement: Supplementary Information — Supplementary Table 1, Supplementary Notes 1-3 and Supplementary References [file ncomms10746-s1.pdf]

Supplementary Table 1: Parameters for the numerical model

Parameter	Value	Units	Source
[actinF]	300	molecules	Assumed
t_{max}	80	min	This publication
k_6	0.002	$molecules^{-1} \cdot min^{-1}$	Estimated
k_7	0.6	$molecules^{-1} \cdot min^{-1}$	{Vogel, 2013 #716}
k_{-6}	0.04	$molecules^{-1} \cdot min^{-1}$	Estimated
k_{-7}	0.4	min^{-1}	{Vogel, 2013 #716}
Cooperativity	2	adimensional	{Kierfeld, 2007 #714}
$[Myo^T]$	0.4	μM	Assumed
$[Rhok^T]$	0.5	μM	Assumed
MM_{Rhok}	2	μM	Assumed
MM_{Flw}	4.5	μM	Assumed
$[Flw^T]_{S6\text{-}S8}$	2.34	μM	This publication
$[Flw^T]_{S9\text{-}S10}$	0.9	μM	Assumed
$[Flw^T]^{lof}$	0.9e-3	μM	Assumed
k^{drug}_{-6}	0.5e-3	$molecules^{-1} \cdot min^{-1}$	This publication
k_2	60	min^{-1}	Estimated
$k_{2\_Constitutively\ active\ Rhok}$	600	min^{-1}	Estimated
k_4	60	min^{-1}	Estimated
Average Myo^p in filament	500	molecules	Cooper and Hausman, *Sinuer Associates, Inc.Sunderland (MA) 2000*
Cell volume	10^{-12}	L	Roskams and Rodgers, Lab Ref, Volume 1, Cold Spring Harbor Laboratory Press (2002)

Supplementary Notes

Supplementary Note 1. Modelling actomyosin basal oscillations

We describe here a theoretical model for the actomyosin oscillations that occur at the basal side of FCs during egg chamber elongation (code available upon request). The model is based on a Gillespie algorithm for stochastic simulations of coupled chemical reactions [1]. Myosin kinetics are modelled as 3 coupled biochemical reactions for the phosphorylation and dephosphorylation of MRLC and the formation of myosin filaments. For simplicity, all potential Rho-associated kinases that phosphorylate MRLC are clustered together in the model as *Rhok*, while all myosin phosphates are clustered as a single variable called *Flw*:

$$Myo + Rhok \xleftrightarrow{k_1, k_{-1}} MyoRock \xrightarrow{k_2} Myo^p + Rhok \qquad (1)$$

$$Myo^p + Flw \xleftrightarrow{k_3, k_{-3}} Myo^p Flw \xrightarrow{k_4} Myo + Flw \qquad (2)$$

$$Myo^p + MyoF_h \xleftrightarrow{k_5, k_{-5}} MyoF_{h+1} \qquad (3)$$

Myo and Myo^p correspond to the concentration of unphosphorylated and phosphorylated forms of myosin, respectively. Phosphorylated myosin molecules then assemble into myofilaments of a few hundred monomers (*myoF*) to then interact with actin filaments (*actinF*) to form actomyosin bundles of n actin and m myosin filaments based on the following interactions:

$$actinF + bundle^i_{n,m} \xrightarrow{n, k_6} bundle^i_{n+1,m} \qquad (4)$$

$$myoF + bundle^i_{n,m} \xrightarrow{n, k_7} bundle^i_{n,m+1} \qquad (5)$$

$$bundle^i_{n,m} \xrightarrow{m, k_{-6}} bundle^i_{n-1,m} + actinF \qquad (6)$$

$$bundle^i_{n,m} \xrightarrow{n, k_{-7}} bundle^i_{n,m-1} + myoF \qquad (7)$$

Equation 4 accounts for the binding of actin filaments above a certain threshold concentration [2], where a given free actin filament interacts with other actin filaments leading to the formation of an actin bundle i formed of n actin and m myosin filaments. This interaction, regulated by

actin binding linker proteins, has been shown to be cooperative, i.e. free actin filaments have increased tendency to aggregate to larger bundles [3]. As a first approximation, we assume quadratic cooperativity in bundle formation (higher cooperativity also reproduces the experimental observations). Equation 5 accounts for the direct interaction of free myosin filaments with actin filaments, either free or in bundle configuration (a free *actinF* is represented in the simulations as a bundle with $n=1$). The rate of this reaction depends on the amount of free myosin available as well as on the amount of actin n in each given bundle i, i.e. larger bundles recruit more myosin molecules than smaller bundles. Equation 6 accounts for the dissociation of actin bundles due to myosin activity [4]. Experiments *in vitro* have shown that myosin II motors control actin bundle/filament turnover in a concentration dependent manner [4]. Based on this, we assume the rate of actin bundle dissociation to be proportional to the amount of myosin filaments in a given i bundle. The released free actin filaments will then be ready to interact via Equation 4 and establish a new bundle j. Finally, Equation 7 illustrates myosin dissociation from a given bundle, with a rate proportional to the amount of myosin filaments in each bundle i. The resulting free myosin molecules can then bind another bundle via Equation 5. For a detailed explanation of the model equations and other relevant information see Supplementary Material. Some of the numerical values for the parameters were estimated from our experimental observations, others were chosen from the literature (see Table 1 and Supplementary Material for a discussion on the selection of model parameters). We find that our model exhibits robust autonomous oscillations within a wide range of parameters, recapitulating FC oscillatory behaviour (Fig.7).

Supplementary Note 2. Calculation of equilibrium concentration of active myosin

Taking into account the mass action law, we obtain from equations 1-2 in the supplement, the following set of chemical equations:

$$\frac{\partial[Myo]}{\partial t} = k_4[Myo^pFlw] - k_1[Myo][Rhok] + k_{-1}[MyoRhok] \quad (8)$$

$$\frac{\partial[MyoRhok]}{\partial t} = k_1[Myo][Rhok] - (k_{-1} + k_2)[MyoRhok] \quad (9)$$

$$\frac{\partial[Myo^pFlw]}{\partial t} = k_3[Myo^p][Flw] - (k_{-3} + k_4)[Myo^pFlw] \quad (10)$$

$$\frac{\partial[Myo^p]}{\partial t} = k_2[MyoRhok] - k_3[Myo^p][Flw] + k_{-3}[Myo^pFlw] \quad (11)$$

Since $[RhoK^T] = [RhoK] + [MyoRhok]$ and $[Flw^T] = [Flw] + [Myo^pFlw]$ (where $[RhoK^T]$ and $[Flw^T]$ correspond to the total concentration of MLC kinases and phosphatases, respectively), equations 8-11 can be rewritten as:

$$\frac{\partial[Myo]}{\partial t} = k_4[Myo^pFlw] - k_1[Myo][Rhok^T] + (k_{-1} + k_1[Myo])[MyoRhok] \quad (12)$$

$$\frac{\partial[MyoRhok]}{\partial t} = k_1[Myo][Rhok^T] - (k_{-1} + k_2 + k_1[Myo])[MyoRhok] \quad (13)$$

$$\frac{\partial[Myo^pFlw]}{\partial t} = k_3[Myo^p][Flw^T] - (k_{-3} + k_4 + k_3[Myo^p])[Myo^pFlw] \quad (14)$$

$$\frac{\partial[Myo^p]}{\partial t} = k_2[MyoRhok] - k_3[Myo^p][Flw^T] + (k_{-3} + k_3[Myo^p])[Myo^pFlw] \quad (15)$$

These constitute a set of four differential equations that can be solved numerically. In addition, if we consider the pseudo-steady state approximation typical for biochemical systems (i.e., the dynamics of intermediate complexes are very fast compared with the other reactions)

$$\frac{\partial\left[MyoRhok\right]}{\partial t}\approx 0 \tag{16}$$

$$\frac{\partial\left[Myo^{p}Flw\right]}{\partial t}\approx 0 \tag{17}$$

With this, we obtain from equations 13-14:

$$\left[MyoRhok\right]=\frac{\left[Myo\right]\left[Rhok^{T}\right]}{\left[Myo\right]+MM_{Rhok}} \tag{18}$$

$$\left[Myo^{p}Flw\right]=\frac{\left[Myo^{p}\right]\left[Flw^{T}\right]}{\left[Myo^{p}\right]+MM_{Flw}} \tag{19}$$

where MM_{Rhok} = $(k_2+k_{-1})/k_1$ and MM_{Flw}= $(k_4+k_{-3})/k_3$ are the Michaelis-Menten constants for kinase and phosphatase, respectively. Substituting equations 18-19 into equations 12, 15, the system can be reduced to a set of two equations:

$$\frac{\partial\left[Myo\right]}{\partial t}=\frac{k_4\left[Myo^{p}\right]\left[Flw^{T}\right]}{\left[Myo^{p}\right]+MM_{Flw}}-k_1\left[Myo\right]\left[Rhok^{T}\right]+\frac{(k_{-1}+k_1\left[Myo\right])\left[Myo\right]\left[Rhok^{T}\right]}{\left[Myo\right]+MM_{Rhok}} \tag{20}$$

$$\frac{\partial\left[Myo^{p}\right]}{\partial t}=\frac{k_2\left[Myo\right]\left[Rhok^{T}\right]}{\left[Myo\right]+MM_{Rhok}}-k_3\left[Myo^{p}\right]\left[Flw^{T}\right]+\frac{(k_{-3}+k_3\left[Myo^{p}\right])\left[Myo^{p}\right]\left[Flw^{T}\right]}{\left[Myo^{p}\right]+MM_{Flw}} \tag{21}$$

That rearranging terms becomes:

$$\frac{\partial\left[Myo\right]}{\partial t}=\frac{k_4\left[Myo^{p}\right]\left[Flw^{T}\right]}{\left[Myo^{p}\right]+MM_{Flw}}+\left[Myo\right]\left[Rhok^{T}\right]\left(\frac{(k_{-1}+k_1\left[Myo\right])\left[Myo\right]\left[Rhok^{T}\right]}{\left[Myo\right]+MM_{Rhok}}-k_1\right) \tag{22}$$

$$\frac{\partial\left[Myo^{p}\right]}{\partial t}=\frac{k_2\left[Myo\right]\left[Rhok^{T}\right]}{\left[Myo\right]+MM_{Rhok}}+\left[Myo^{p}\right]\left[Flw^{T}\right]\left(\frac{(k_{-3}+k_3\left[Myo^{p}\right])\left[Myo^{p}\right]\left[Flw^{T}\right]}{\left[Myo^{p}\right]+MM_{Flw}}-k_3\right) \tag{23}$$

that becomes:

$$\frac{\partial[Myo]}{\partial t}=\frac{k_4[Myo^p][Flw^T]}{[Myo^p]+MM_{Flw}}-\frac{k_2[Myo][Rhok^T]}{[Myo]+MM_{Rhok}} \quad (24)$$

$$\frac{\partial[Myo^p]}{\partial t}=\frac{k_2[Myo][Rhok^T]}{[Myo]+MM_{Rhok}}-\frac{k_4[Myo^p][Flw^T]}{[Myo^p]+MM_{Flw}} \quad (25)$$

This satisfies the relation $\partial[Myo]/\partial t=-\partial[Myo^p]/\partial t$, since $[Myo^T]=[Myo]+[Myo^p]$. Therefore, the amount of phosphorylated myosin can be described by a single differential equation:

$$\frac{\partial[Myo^p]}{\partial t}=\frac{k_2([Myo^T]-[Myo^p])[Rhok^T]}{[Myo^T]-[Myo^p]+MM_{Rhok}}-\frac{k_4[Myo^p][Flw^T]}{[Myo^p]+MM_{Flw}} \quad (26)$$

The dynamics of biochemical reaction is typically in the order of seconds, which is much faster than the dynamics of the oscillations observed experimentally (on the order of a few minutes). Therefore, the time scales of the two processes can be separated and we can safely assume that the amount of active myosin when compared to the dynamics of the binding-dissociation of the actomyosin network is in equilibrium ($[Myo^p{}_{eq}]$). Under these conditions, $\partial[Myo]/\partial t\approx 0$, and equation 26 becomes:

$$\begin{aligned} &k_2([Myo^T]-[Myo^p_{eq}])[Rhok^T]([Myo^p_{eq}]+MM_{Flw})=\ldots \\ &\ldots k_4[Myo^p_{eq}][Flw^T]([Myo^T]-[Myo^p_{eq}]+MM_{Rhok}) \end{aligned} \quad (27)$$

This is a quadratic equation that can be solved to obtain a solution for the concentration of active myosin in equilibrium as:

$$[Myo^p_{eq}]=\frac{-B\pm\sqrt{B^2-4W[Myo^T][MM_{Flw}](1-W)}}{2(1-W)} \quad (28)$$

with $W=k_2[Rhok^T]/(k_4[Flw^T])$ and $B= W([Myo^T]-[Flw^T])- [Flw^T]-[MM_{Rhok}]$. In the context of our simulation, the value $[Myo^p_{eq}]$ is calculated for a given constant concentration of Flw and Rhok, and assumed as constant during the simulation.

Supplementary Note 3. Parameter estimation and Numerical solution of the model equations

For the sake of simplicity, each reaction in the model is a combination of multiple molecular interactions between actin filaments, myosin, ATP and bundling proteins. This simplification results in a complex mapping of the reaction rates used to experimentally measured reaction rates. When possible, parameter values were chosen based on experimental estimations from the literature. When not available, parameters are selected based on qualitative analysis of the experimental behavior. Parameter selection for each reaction rate is explained below. Equations are solved using a Gillespie algorithm for stochastic simulations in the following way: for each interaction of the model, the reaction that takes place is selected stochastically with a probability that depends on its rate. Once a particular reaction is selected, the particular bundle that reacts is also set stochastically with a different probability for each of the seven reactions, in the following way:

Reactions 1-2: Myosin phosphorylation. The dynamics of phosphorylation and dephosphorylation of myosin is considered much faster than the dynamics of binding and dissociation of bundles, therefore the amount of active myosin is calculated based on equation 28 and is considered constant throughout the simulation and calculated as explained in the previous Appendix I.

Due to lack of experimental values, concentrations and chemical constants for the reactions of phosphorylation and dephosphorylation of myosin are chosen to match the experimental

conditions of 5X increase in active [Myo^P] in mutants compared to wild type situation. For the sake of simplicity, we assume a constant concentration of myosin in both wild type and mutant conditions, assuming that the main result of the *flw* mutation is simply to increase levels of active myosin. The difference in concentration between S9-S10 and mutants in [Flw^T] is higher than 3 orders of magnitude, to match the experimental conditions. Parameters for all constants and concentrations can be found in Supplementary Table 1. Values of the concentrations and Michaelis-Menten constants are expressed in μM. To convert these values to number of molecules (required for the aggregation-dissociation simulation), we assume a cell volume of 10^{-12} L.

Reaction 3: Myosin polymerization into myofilaments. Dynamics of myosin and actin polymerization into filaments is of the order of a few seconds, with an average myofilament length of the order of a few hundred active myosin molecules. If we assume that equilibrium is reached in a time scale of an order of magnitude faster than the dynamics of binding and dissociation of the bundles, we can safely assume that the amount of myofilaments formed is simply based on the amount of active myosin present. Therefore, the amount of myofilaments for each simulation is simply calculated by dividing the amount of active myosin by the average number of myosin molecules in a myofilament (assumed to be 500). We therefore assumed that phosphorylated myosin molecules assemble into myo-filaments with an average of 500 molecules [5] to finally obtain the final number of filaments of active myosin-filaments that interact with the actin filaments via equations 4-7. Estimation of the amount of filaments of actin and myosin at a given time in the cell is not available. Since our model solves the system of equations for each filament of myosin and actin, it is convenient to use low values of total actin and myosin filaments to reduce the computational cost of the simulations. Oscillatory behaviour can be achieved for values of actin filaments between 100 and 1000.

Reaction 4: Actin-Actin binding. The process of actin filament binding into bundles requires the action of actin crosslinking proteins, such as fascin, villin and fimbrin [6, 7]. This provides the cooperative binding of actin filaments into bundles. This complex molecular process involving several molecular interactions is condensed in a single reaction with reaction rate k_6. Estimation of this reaction rate is not available experimentally in the context of follicle cells, so we assume a value that results in bundle formation with a dynamic equivalent to experimental data (of the order of minutes). The probability of actinF binding is proportional to the total amount of free ActinF. The probability for a particular free actinF to associate to a particular bundle depends on the amount of actinF in each bundle with quadratic cooperativity (higher cooperativity also reproduces the experimental observations).

Reaction 5: Myosin-actin binding. The work of Vogel et al. estimated a rate of k_7=30 s^{-1} [8], which to be used in our model needs to be divided by the total actin in the system and converted to minutes, giving us the value of $k_7 = 0.6\ min^{-1}$. The probability of binding between myosinF and actinF is proportional to the amount of free myosinF in the system. The probability for a myosin filament to attach to a given actin bundle is proportional to the amount of actin filaments in the bundle.

Reaction 6: Actin-Actin dissociation. Dissociation of a given actin filament involves an increase of tension in the bundle due to mechanical myosin motor action, followed by the release of actin due to the increase tension in the bundle. Our model combines the reaction rates of these two processes in a single reaction with a rate k_{-6}. Estimation of potential values of k_{-6} cannot be extracted from the literature. We estimate this value so the rate of the dissociation is on the range of the forward reaction of actin-actin binding. The probability for actinF release from the bundles is proportional to the total amount of myosinF and actinF in all bundles. The probability for a particular actinF to get released from a given bundle is

proportional to the amount of F-myosin in this particular bundle.

Reaction 7: Myosin-actin release. The release of myosin has been previously modeled as three consecutive reactions: release of ADP, binding to ATP and final dissociation from the actin filament [8]. Our model combines these three reactions with rate constants on the order of 2×10^3 s^{-1}, into one single reaction. We estimate the combined reaction rate constant for the myosin dissociation to counteract the forward reaction. These values are around $0.1 < k^{-7} < 1.5$. For the simulations used here, we use a value of 0.3 min^{-1}. The probability of the release reaction of myosinF from the bundles is proportional to the amount of the total myosinF attached to actinF. The probability of release of myosinF from a particular bundle is proportional to the amount of myosinF attached to each particular bundle.

Supplementary References

1. Gillespie, D.T. Exact stochastic simulation of coupled chemical reactions. *The Journa of Physical Chemistry* **81**, 2340-2361 (1977).
2. Tang, J.X. & Janmey, P.A. Two distinct mechanisms of actin bundle formation. *Biol Bull* **194**, 406-408 (1998).
3. Kierfeld, J., Kraikivski, P., Kuhne, T. & Lipowsky, R. Cooperative behaviour of semiflexible polymers and filaments. *Traffic and Granular Flow ´05, ed. by A. Schadschneider et al. (Springer, Berlin, 2007)*, 239-249 (2007).
4. Haviv, L., Gillo, D., Backouche, F. & Bernheim-Groswasser, A. A cytoskeletal demolition worker: myosin II acts as an actin depolymerization agent. *J Mol Biol* **375**, 325-330 (2008).
5. Cooper, G., M. & Hausman, R.E. The Cell: A Molecular Approach. *Sinuer Associates, Inc.Sunderland (MA) 2000* **Sixth Edition** (2013).
6. Jansen, S. *et al.* Mechanism of actin filament bundling by fascin. *J Biol Chem* **286**, 30087-30096 (2011).
7. Pollard, T.D. & Cooper, J.A. Actin and actin-binding proteins. A critical evaluation of mechanisms and functions. *Annu Rev Biochem* **55**, 987-1035 (1986).
8. Vogel, S.K., Petrasek, Z., Heinemann, F. & Schwille, P. Myosin motors fragment and compact membrane-bound actin filaments. *Elife* **2**, e00116 (2013).
